# Stress-induced TRAILR2 expression overcomes TRAIL resistance in cancer cell spheroids

**DOI:** 10.1038/s41418-020-0559-3

**Published:** 2020-05-20

**Authors:** Daniela Stöhr, Jens O. Schmid, Tobias B. Beigl, Alexandra Mack, Daniela S. Maichl, Kai Cao, Beate Budai, Gavin Fullstone, Roland E. Kontermann, Thomas E. Mürdter, Stephen W. G. Tait, Cathrin Hagenlocher, Nadine Pollak, Peter Scheurich, Markus Rehm

**Affiliations:** 1 Institute of Cell Biology and Immunology, University of Stuttgart, 70569 Stuttgart, Germany; 2 Dr. Margarete Fischer-Bosch Institute of Clinical Pharmacology and University of Tuebingen, 70376 Stuttgart, Germany; 3 Department of Laboratory Medicine, Robert-Bosch-Hospital, 70376 Stuttgart, Germany; 4 Cancer Research UK Beatson Institute and Institute of Cancer Sciences, University of Glasgow, Garscube Estate, Switchback Road, Glasgow, G61 1BD UK; 5 Stuttgart Research Center Systems Biology, University of Stuttgart, 70569 Stuttgart, Germany; 6 Department of Physiology & Medical Physics, Royal College of Surgeons in Ireland, Dublin D2, Ireland; 7 Centre for Systems Medicine, Royal College of Surgeons in Ireland, Dublin D2, Ireland

**Keywords:** Cancer microenvironment, Tumour heterogeneity

## Abstract

The influence of 3D microenvironments on apoptosis susceptibility remains poorly understood. Here, we studied the susceptibility of cancer cell spheroids, grown to the size of micrometastases, to tumor necrosis factor-related apoptosis-inducing ligand (TRAIL). Interestingly, pronounced, spatially coordinated response heterogeneities manifest within spheroidal microenvironments: In spheroids grown from genetically identical cells, TRAIL-resistant subpopulations enclose, and protect TRAIL-hypersensitive cells, thereby increasing overall treatment resistance. TRAIL-resistant layers form at the interface of proliferating and quiescent cells and lack both TRAILR1 and TRAILR2 protein expression. In contrast, oxygen, and nutrient deprivation promote high amounts of TRAILR2 expression in TRAIL-hypersensitive cells in inner spheroid layers. COX-II inhibitor celecoxib further enhanced TRAILR2 expression in spheroids, likely resulting from increased ER stress, and thereby re-sensitized TRAIL-resistant cell layers to treatment. Our analyses explain how TRAIL response heterogeneities manifest within well-defined multicellular environments, and how spatial barriers of TRAIL resistance can be minimized and eliminated.

## Introduction

The cytokine tumor necrosis factor (TNF)-related apoptosis-inducing ligand (TRAIL) is a member of the TNF superfamily and can trigger caspase-8-dependent apoptosis in transformed cells by binding and activating two receptors, TRAIL receptor 1 (TRAILR1) and TRAIL receptor 2 (TRAILR2) [1, 2]. Various TRAIL-based therapeutics have been developed in the recent years, including aggregated TRAIL variants, TRAILR-binding agonistic antibodies, and multivalent antibody-TRAIL fusion proteins that show increased stability and activity in comparison to soluble recombinant human TRAIL (dulanermin) [1, 3]. Following binding, TRAILRs oligomerize into the death inducing signaling complex (DISC), which also comprises of Fas-associated protein with death domain (FADD). Procaspase-8 binds to FADD and forms filaments in which procaspase-8 is auto-proteolytically cleaved and activated [4–7]. By competing with procaspase-8 for binding to the elongating filaments, cellular FLICE-like inhibitory protein (cFlip), a procaspase-8 homolog lacking protease activity, can inhibit the full activation and processing of procaspase-8 [8]. High amounts of active caspase-8 and/or low concentrations of effector caspase inhibitor XIAP allow direct activation of effector caspase-3 and apoptosis execution [9, 10]. However, in most cells caspase-8 must cleave the BH3-only protein Bid to truncated Bid (tBid), which translocates to the outer mitochondrial membrane to induce the formation of Bak and Bax pores, which induces apoptosome-dependent caspase-3 activation and apoptosis execution. The threshold for whether tBid can induce Bax/Bak pores or not is set by antiapoptotic members of the Bcl-2 protein family [11].

First generation TRAILR agonists were described as potent apoptosis inducers in cellular models of various cancers, however, therapeutic candidates so far performed rather disappointingly. In phase I and phase II clinical trials, only few patients responded to treatment with dulanermin or TRAILR-specific agonistic antibodies, such as Mapatumumab (anti-TRAILR1) and Conatumumab (anti-TRAILR2) [3, 12]. Besides insufficient receptor oligomerisation, an aspect that has been addressed by the development of multivalent antibody-TRAIL fusion proteins of superior potency [13, 14], in vivo half times of dulanermin, lying in the range of hours, also considerably limited clinical efficacy [12]. For TRAIL-based treatments to be effective, it is therefore crucial that cancer cell killing can be achieved rapidly and that TRAIL-based therapeutics encounter TRAIL responsive cells also in deeper layers of solid tumors.

Compared with conventional adherent cell cultures, cancer cell spheroids, grown to sizes of micrometastases, and resembling cellular interactions and architectures found in avascular tumor tissues, offer more advanced but nevertheless well controllable experimental models suited to assess drug treatment efficacies and to study the modulation of cellular responsiveness to treatment as a consequence of the spheroid microenvironment [15]. Here, we therefore studied the TRAIL responsiveness of spheroids and discovered that the spheroid microenvironment gives rise to layers of TRAIL-resistant cells. These TRAIL-resistant cells prevent apoptosis induction in TRAIL-hypersensitive cells that develop deeper within the spheroids, and thereby increase overall TRAIL resistance. This can be counteracted by intensifying microenvironmental stress that drives TRAILR2 expression.

## Materials and methods

### Reagents and antibodies

QVD (Q-VD-OPH) and celecoxib were purchased from Selleckchem (Houston, TX, USA). IZI1551 (TRAIL) was produced as described before [14]. Annexin V-EGFP was produced in-house. DAPI (4′,6-diamidin-2-phenylindol) was obtained from Thermo Fisher Scientific (Waltham, MA, USA). DMSO was purchased from Carl Roth (Karlsruhe, Germany). The following antibodies were used for flow cytometry: mouse monoclonal TRAILR1 (MAB347, R&D Systems, Wiesbaden-Nordenstadt, Germany), mouse monoclonal TRAILR2 (MAB6311, R&D Systems, Wiesbaden-Nordenstadt, Germany), mouse monoclonal FADD (ab119059, Abcam, Cambridge, UK), mouse monoclonal Flip (7F10, Enzo Life Sciences, Lorrach, Germany), rabbit monoclonal procaspase-8 (ab32125, Abcam, Cambridge, UK), rabbit monoclonal Ki67 (D3B5, Cell Signaling, Danvers, MA, USA), purified mouse IgG1 *κ* istotype control and purified mouse IgG2b *κ* istotype control (BD Biosciences, Heidelberg, Germany), rabbit isotype control (DA1E, Cell Signaling, Danvers, MA, USA), and goat antirabbit Alexa Flour 647 (IgG (H + L) highly cross-adsorbed, A-21245), goat antimouse Alexa 488 (IgG (H + L) highly cross-adsorbed, A-11029), goat antirabbit Alexa 488 (IgG (H + L) highly cross-adsorbed, A-11008) from Thermo Fisher Scientific (Waltham, MA, USA). The following antibodies were used for western blotting: rabbit monoclonal LC3B (D11, Cell Signaling, Danvers, MA, USA), mouse monoclonal α-tubulin (DM1A), rabbit monoclonal COX IV (3E11), mouse monoclonal GAPDH (D4C6R), mouse monoclonal Hif-1α (D5F3M), rabbit monoclonal TRAILR1 (D9S1R), rabbit monoclonal TRAILR2 (D4E9), rabbit polyclonal FADD, rabbit monoclonal Flip (D16A8), rabbit monoclonal cFlip-L/-S (D5J1E), rabbit monoclonal Caspase-8 (D35G2), mouse monoclonal Caspase-8 (1C12), rabbit monoclonal XIAP (D2Z8W), rabbit polyclonal cleaved Caspase-3 and rabbit monoclonal Caspase-3 (8G10) from Cell Signaling (Danvers, MA, USA), mouse monoclonal CHOP (GADD 153 (B-3): sc-7351, Santa Cruz, Santa Cruz, CA, USA), antimouse IgG HRP-linked antibody and antirabbit IgG HRP-linked antibody from Cell Signaling (Danvers, MA, USA), IRDye® 800CW goat antimouse IgG secondary antibody and IRDye® 680RD goat antirabbit IgG secondary antibody from LI-COR Biosciences (Lincoln, NE, USA). The following antibodies were used for immunohistochemistry: mouse monoclonal Hif-1α (BD Biosciences, Heidelberg, Germany), rabbit monoclonal thymidine kinase 1 (EPR3191, Abcam, Cambridge, UK), goat polyclonal TRAILR1 (sc-6823, Santa Cruz, Santa Cruz, CA, USA), mouse monoclonal TRAILR2 (TR2.21, AdipoGen Life Science, Liestal, Switzerland), bridging antibody rabbit antigoat (Gentaur, Aachen, Germany). For immunofluorescence staining of Ki67, mouse monoclonal Ki67 (8D5) from Cell Signaling (Danvers, MA, USA) and goat antimouse Alexa 647 (IgG (H + L) highly cross-adsorbed, A-21236, Thermo Fisher Scientific, Waltham, MA, USA) was used.

### Cell culture

NCI-H460 cells were purchased from ATCC (Manassas, VA, USA), HCT116 cells were obtained from the Banca Biologica e Cell Factory of the IRCCS Azienda Ospedaliera Universitaria San Martino in Genoa (ICLC HTL95025). Simian virus 40 large T-immortalized murine embryonic fibroblasts from TNFR1/TNFR2 double knockout mice that stably express either human TRAILR1 or human TRAILR2 (MEF-hT1; MEF-hT2) were kindly provided by Dr Simon Neumann (University of Stuttgart, Germany). TRAILR1 deficient HCT116 cells (HCT116 T1 k/o), TRAILR1 deficient NCI-H460 cells (NCI-H460 T1 k/o), TRAILR2 deficient HCT116 cells (HCT116 T2 k/o) and TRAILR2 deficient NCI-H460 cells (NCI-H460 T2 k/o) were generated by CRISPR/Cas9-based gene targeting. HCT116 cells were transfected with the guide RNA containing vector via lipofectamine while NCI-H460 cells underwent lentiviral transduction. Oligos coding for the guide RNA were ordered from biomers.net. Guide RNA against TRAILR1 in HCT116: 5′-CACCgCGTGGTTCAATCCTCCCCG-3′ (forward), 5′-AAACCGGGGAGGATTGAACCACGC-3′ (revers). Guide RNA against TRAILR2 in HCT116: 5′-CACCGCAGAACGCCCCGGCCGCTT-3′ (forward), 5′- AAACAAGCGGCCGGGGCGTTCTGC-3′ (reverse). Both oligos were annealed and ligated into pSpCas9(BB)-2A-GFP ((PX458), (#48138), Addgene, Watertown, MA, USA). Two days after cell transfection, GFP positive clones were sorted (BD5 FACSAriaTM III, BD Biosciences, Heidelberg, Germany) and analyzed for successful TRAILR knockout by flow cytometry and western blotting after 2–3 weeks of cultivation. Guide RNA against TRAILR1 in NCI-H460: 5′-CACCGAGTACATCTAGGTGCGTTCC-3′ (forward), 5′-AAACGGAACGCACCTAGATGTACTC-3′ (revers). Guide RNA against TRAILR2 in NCI-H460: 5′-CACCGATAGTCCTGTCCATATTTGC-3′ (forward), 5′-AAACGCAAATATGGACAGGACTATC-3′ (revers). Both oligos were annealed and ligated into lentiCRISPRv2 (#52961, Addgene, Watertown, MA, USA). To produce the lentiviral particles for the transduction of NCI-H460 cells, HEK293T cells were transfected with the vector containing the guide RNA together with a vector for viral packaging (psPAX2, #12260, Addgene, Watertown, MA, USA) and for the viral envelope (pCMV-VSV-G, #8454, Addgene, Watertown, MA, USA) using lipofectamine. After selection with puromycin, the pool of cells was sorted for the absence of TRAILR expression using primary and Alexa 488 labeled secondary antibodies (mouse monoclonal TRAILR1 (MAB347, R&D Systems, Wiesbaden-Nordenstadt, Germany), goat antimouse Alexa 488 (IgG (H + L) highly cross-adsorbed, A-11029, Thermo Fisher Scientific, Waltham, MA, USA)). Obtained single clones were analysed for successful TRAILR knockout by flow cytometry and western blotting after 2–3 weeks of cultivation.

All cell lines were grown in Roswell Park Memorial Institute medium (RPMI, Thermo Fisher Scientific, Gibco, Waltham, MA, USA) with 10% FCS (fetal calf serum, PAN-Biotech, Aidenbach, Germany) except for NCI-H460 cells that were cultured with 5% FCS. For generation of spheroids, cells were seeded into Terasaki multiwell plates (100 cells/well, Greiner Bio-One, Frickenhausen, Germany) and placed in humid chambers in the incubator. After 3 days of cultivation, spheroids were transferred to agarose-coated 96-well plates (F-bottom, Greiner Bio-One, Frickenhausen, Germany). Medium was changed every second to third day. To induce hypoxia, 2D-cultured cells were placed in a hypoxia chamber (O_2_ Control InVitro Glove Box, Coy Laboratory Products, Grass Lake, MI, USA; 1% oxygen, 5% CO_2_, 37 °C, and 96% relative humidity). At the end of experiments, cells were harvested inside of the hypoxia chamber and immediately put on ice. All articles and media were gas equilibrated within the hypoxic chamber at least 24 h prior to usage. For experiments with nutrient deprivation, 2D-cultivated cells were grown in RPMI containing 10% FCS and either 2, 1, 0.5, or 0.25 mg/ml glucose (Carl Roth, Karlsruhe, Germany) or RPMI medium without FCS and with 2 mg/ml glucose. All cell lines were regularly tested for mycoplasma infection and their authenticity was verified by STR profiling.

### Cell death measurements

Cells were harvested or spheroids were dissociated with trypsin/EDTA (Thermo Fisher Scientific, Gibco, Waltham, MA, USA), stained with Annexin V-EGFP in Annexin V-EGFP binding buffer (BD Biosciences, Heidelberg, Germany) for 10 min at room temperature, and then analyzed by flow cytometry (MACSQuant Analyzer 10, Miltenyi Biotec, Bergisch Gladbach, Germany).

### qPCR

RNA was extracted using the RNeasy Plus Mini Kit (QIAGEN, Hilden Germany), following the manufacturer’s instruction. RNA concentrations were measured on a NanoDrop Spectrophotometer ND-1000, (Thermo Fisher Scientific). For the generation of cDNA, RNA extracts were diluted with RNase-free water (1:10) and RNA (100 ng) was mixed with 2 μl wipe out buffer (QuantiTect Reverse Transcriptase Kit, QIAGEN, Hilden, Germany). After incubation at 42 °C for 2 min, reverse transcriptase (RTase), RTase buffer, and RTase primers were added and samples were incubated, first for 15 min at 42 °C and then 3 min at 95 °C, following the manufacturer’s instructions (QuantiTect Reverse Transcriptase Kit, QIAGEN). cDNA concentrations were determined prior to qPCR. Primers were designed with Primer-BLAST from NCBI and ordered from biomers net (Ulm, Germany). Primers were mixed with cDNA (100 ng) and DyNAmo ColorFlash SYBR Green qPCR Mix (2×) following the manufacturer’s instructions (DyNAmo ColorFlash SYBR Green qPCR Kit, Biozym, Hessisch Oldendorf, Germany) and reactions were measured on a qPCR Cfx96 device (Biorad, Hercules, CA, USA). Fold change expression of mRNA was calculated by the ΔΔCt method using the CFX Manager Software (Biorad, Hercules, CA, USA). The following qPCR primers were used: GAPDH: 5′-CCCCTTCATTGACCTCAACTA-3′ (forward primer) 5′-CGCTCCTGGAAGATGGTGAT-3′ (reverse primer); TRAILR1: 5′-GGTTGTTCCGTTGCTGTTGG-3′ (forward primer), 5′-CCAGAAACACACCCTGTCCAT-3′ (reverse primer); TRAILR2-L: 5′-CCCTGTTCTCTCTCAGGCATC-3′ (forward primer), 5′-CAGGTCGTTGTGAGCTTCTGT-3′ (reverse primer); TRAILR2-S: 5′-GTCCACAAAGAATCAGGCATC-3′ (forward primer), 5′- CCAGGTCGTTGTGAGCTTCT-3′ (reverse primer); TRAILR4: 5′-GGAGACAGTGACCACCATCC-3′ (forward primer), 5′-CGCCGGAAAAGGACTCTGT-3′ (reverse primer).

### Flow cytometric analysis of cellular protein amounts

For measurements of cell surface receptor amounts, cells were suspended in cold PBA (1 × 10^5^ cells per sample, PBS + 0.05% (w/v) bovine serum albumin (BSA, Sigma-Aldrich, Munich, Germany) + 0.02% (w/v) NaN_3_ (Carl Roth, Karlsruhe, Germany) in ddH_2_0) containing primary antibody (100 µl/sample, on ice). After 1 h incubation, cells were washed with PBA and resuspended in PBA containing secondary antibody (on ice, 1 h). Thereafter, cells were washed with PBA and analyzed (MACSQuant Analyzer 10, Miltenyi Biotec, Bergisch Gladbach, Germany). To combine surface amount measurements with intracellular Ki67 measurements, cells stained for surface receptors were washed with PBS and fixed with 4% paraformaldehyde (4% PFA (v/v) in PBS, Merck, Darmstadt, Germany) for 10 min at room temperature in the dark. After two times washing with PBS, the pellet was resuspended in ice-cold PBS (10 μl) and 90 μl methanol (100%, −20 °C, Carl Roth, Karlsruhe, Germany) was added. Following 30 min of incubation on ice, 50 μl PBA containing 5% (w/v) BSA (washing solution) was added prior to centrifugation at 500 g for 5 min at room temperature. After two more washing steps, cell pellets were suspended in 100 μl washing solution containing primary antibody, and cells were incubated for 1 h at room temperature in the dark. After washing, cells were incubated with 100 μl washing solution containing secondary antibody for 1 h at room temperature in the dark. After a final wash, cells were resuspended in PBA, and fluorescence was measured (MACSQuant Analyzer 10, Miltenyi Biotec, Bergisch Gladbach, Germany). All flow cytometric data were analyzed by FlowJo 7.6.1 (Tree Star Inc.) or MACSQuantify (MACS Miltenyi Biotec, Bergisch Gladbach, Germany).

### Preparation and staining of cryosections

Spheroids were collected in a 1.5 ml reaction tube, washed with PBS and fixed with 4% PFA for 10 min at room temperature, followed by incubation with sucrose (30% (w/v) in PBS; Carl Roth, Karlsruhe, Germany) for 48 h at 4 °C. After removal of sucrose, 1 ml of Tissue Freezing Medium (Tissue-Tek O.C.T Compound (TTEK); A. Hartenstein, Würzburg, Germany) was added. Spheroids were stored at −20 °C until usage. To prepare cryosections, samples were mounted on precooled sample holders and cut into 10 μm slices (CM30505 cryostat, Leica Biosystems, Wetzlar, Germany). Sections were mounted on Polysine Microscope Adhesion Slides (Thermo Fisher Scientific, Waltham, MA, USA). After thawing, cryosections were fixed with 100% methanol (Carl Roth, Karlsruhe, Germany) for 10 min at −20 °C, washed with PBS and blocked with a BSA solution (5% (w/v) in PBS) for 10 min at room temperature. After washing again with PBS, sections were incubated with 100 μl peroxidase blocking reagent (Dako, Agilent Technologies, Santa Clara, CA, USA) for 5 min at room temperature and, after washing, primary antibody (diluted in Antibody Diluent (Dako, Agilent Technologies, Santa Clara, CA, USA)) was added for 1 h at room temperature. Next, sections were washed with PBS and either incubated with 100 μl of a peroxidase labeled polymer, binding to rabbit or mouse derived primary antibodies (Dako, Agilent Technologies, Santa Clara, CA, USA) for 30 min at room temperature, or in case of the TRAILR1 antibody, which derived from goat, samples were treated with 100 μl of a bridging antibody (rabbit antigoat IgG, IgM, IgA, Gentaur, Aachen, Germany) for 30 min at room temperature. Next, sections were washed again with PBS, incubated with 100 μl of a substrate chromogen solution (chromogen 3, 3′-Diaminobenzidine 1:50 in substrate buffer, Dako, Agilent Technologies, Santa Clara, CA, USA) for 10 min and washed under running water for 3 min. Cell nuclei were stained with hematoxylin solution (Merck, Darmstadt, Germany). Afterwards, cryosections were treated with ethanol solutions (70%, 90%, 100%, each 3 min) and incubated with the xylene substitute Neo-Clear (Merck, Darmstadt, Germany) for 3 min. Stained cryosections were mounted using Neo-Mount (Merck, Darmstadt, Germany).

### Preparation and staining of paraffin sections

Spheroids were washed once with PBS and transferred into an agarose bedding (4% (w/v) agarose in ddH_2_O). Spheroids were then stained with hematoxylin solution (Merck, Darmstadt, Germany) to identify spheroid locations. Spheroids were then incubated with 4% PFA solution for 10 min at room temperature and, after removal of supernatant, covered with 1% agarose. Agarose cores were transferred into 4% PFA in embedding cassettes. Samples were dehydrated and embedded in paraffin. In total, 3 μm slices were cut with a rotary microtome (RM 2255 Mikrotom, Leica Biosystems, Wetzlar, Germany) and sections were transferred to glass slides before they were incubated at 56 °C overnight, prior to storage. Sections were deparaffinized with Neo-Clear solution (Merck, Darmstadt, Germany) for 30 min at room temperature and rehydrated with decreasing concentrations of ethanol (100%, 96%, 70%) and H_2_O (3 min each). Next, samples were incubated with Dako target retrieval solution pH 6 (Dako, Agilent Technologies, Santa Clara, CA, USA) for 30 min inside a steam cooker and afterwards washed two times for 3 min with 1 × TBST (Tris-buffered saline with 0.1% (v/v) Tween-20, Carl Roth Karlsruhe, Germany). Treatment with peroxidase blocking reagent (Dako, Agilent Technologies, Santa Clara, CA, USA) and staining steps were conducted as described above.

### Immunostaining of cryosections

Sections were washed with PBS, followed by fixation for 10 min at room temperature with 4% PFA and two washing steps with PBS for 5 min. After permeabilisation with 0.1% (v/v) Triton X-100 (Carl Roth, Karlsruhe, Germany) in PBS at room temperature for 10 min, sections were treated with blocking solution (5% (v/v) FCS and 0.1% (v/v) Triton X-100 in PBS) at room temperature for 30 min, and then incubated with the primary antibodies for 1 h at room temperature. Sections were washed twice and incubated with the Alexa 647-conjugated secondary antibodies for 1 h at room temperature. After two washes, DNA was stained using DAPI (1 μg/ml in PBS, 10 min, room temperature). Following two washes in PBS, coverslips were mounted on top of glass slides using Fluoromount-G (Southern Biotechnology Associates, Birmingham, AL, USA).

### Analysis of sections

Cryosections were analyzed using a laser scanning microscope (LSM 710, Carl Zeiss, Germany) and Zen 2010 black edition (Carl Zeiss, Germany). Alternatively, stained spheroids sections were imaged using a bright field slide scanner (Leica SCN400, Leica Microsystems, Wetzlar, Germany), and images were analyzed using Definiens Tissue Studio 64 software (Definiens AG, Munich, Germany). Photoshop Elements 10 software (Adobe Systems, San José, CA, USA) was used to determine percentages of stained cells and their intensities in spheroid layers.

### Western blotting

2D-cultured cells and spheroids were washed with ice-cold PBS before they were incubated on ice in RIPA buffer (150 mM NaCl, 0.1% Triton X-100, 0.5% sodium deoxycholat, 0.1% sodium dodecyl sulfate (SDS), 50 mM Tris in ddH_2_O, pH 8; all chemicals from Carl Roth, Karlsruhe, Germany) for 5 min, sonicated (6 pulses, Bandelin Sonopuls HD 200, BANDELIN electronic GmbH & Co. KG, Berlin, Germany), and incubated for another 5 min on ice. Cellular debris was removed by centrifugation at 20,000 g for 20 min. Protein concentrations were quantified by Bradford assay. Equal amounts of proteins were supplemented with 5 × Laemmli sample buffer (10% SDS, 312.5 mM Tris pH 6.8, 25% β-mercaptoethanol, 25% glycerin, 0.05% bromphenol blue, all chemicals were purchased from Carl Roth, Karlsruhe, Germany) and heated to 95 °C for 5 min. Proteins were separated on Nu-Page 4–12% Bis-Tris gels (Invitrogen, Carlsbad, CA, USA) and transferred to nitrocellulose membranes using an iBlot 2 gel transfer device (Invitrogen, Carlsbad, CA, USA). After 1 h blocking with blocking reagent (Roche Diagnostics, Mannheim, Germany) diluted in TBST (1%) the membranes were incubated with primary antibodies (diluted in TBST with 0.5% blocking reagent) overnight. After washing with TBST, membranes were incubated either with an HRP-coupled secondary antibody (diluted in TBST with 0.5% blocking reagent) or an IRDye-conjugated secondary antibody (diluted in TBST with 0.5% blocking reagent) for 1 h at room temperature. Following three further washing steps, proteins were detected by either directly measuring fluorescence with an infrared imager (LI-COR Odyssey, LI-COR Biosciences, Lincoln, NE, USA) or incubating the membrane with an HRP substrate (SuperSignal West Pico ECL Substrate/SuperSignal West Dura Extended, Thermo Scientific Pierce Protein Biology, Waltham, MA, USA; Luminata Forte Western HRP Substrate, Merck Millipore, Burlington, MA, USA) and detecting the signals with an ECL imager (Amersham Imager 600, GE Healthcare, Freiburg, Germany GmbH).

### Calculation of the coefficient of drug interaction (CDI)

CDIs were calculated according to Cao and Zhen [16]. ${\mathrm{CDI}} = \frac{{E\left( {AB} \right)}}{{E\left( A \right) \ast E\left( B \right)}}$ where *E*(*A*), *E*(*B*), and *E*(*AB*) are the ratios, in percentage of surviving cells, following treatment with drug *A*, *B*, and *A* + *B*, respectively, in comparison to control groups. CDIs < 1 indicate synergism.

### Statistical analysis

Statistical analysis was performed using GraphPad Prism 5 (GraphPad Software, San Diego, CA, USA). Data are shown as mean values plus and minus the standard deviation (SD) or standard error of the mean (SEM) as stated in the figure legends. Statistical significance of differences between groups was verified using the stated significance tests. Significance level were denoted with asterisks: *p ≤ 0.5; **p ≤ 0.01; ***p ≤ 0.001. Means were calculated from independently performed experiments.

## Results

### TRAIL-resistant cell layers protect TRAIL-sensitive cells within cancer cell spheroids

**Fig. 1:**
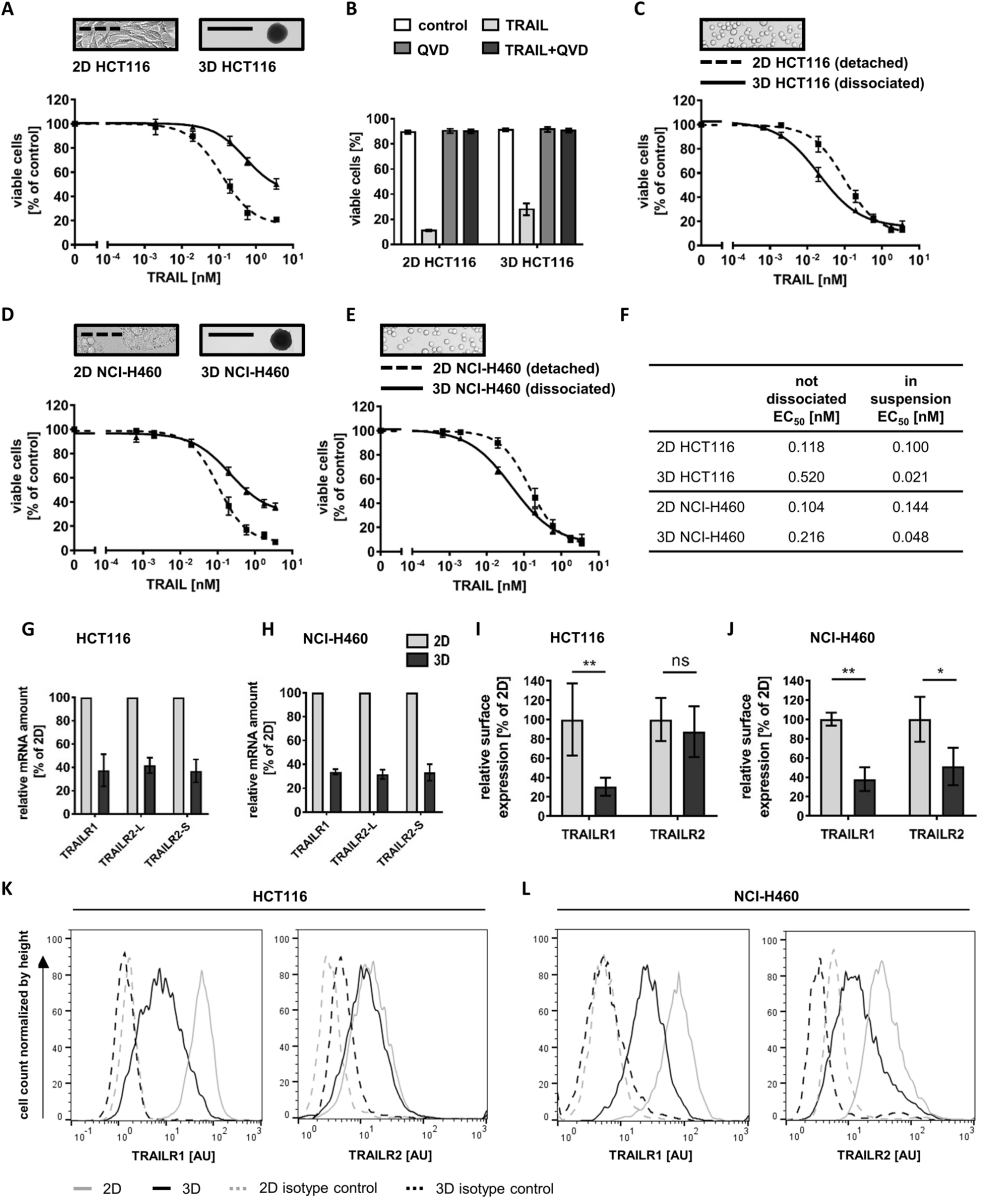
TRAIL-resistant cell layers protect TRAIL-sensitive cells within tumor cell spheroids. **a**, **d** Cell viability in HCT116 and NCI-H460 cells, grown in 2D or as spheroids (day 11), following 6 h of treatment with TRAIL. The loss of viability was measured flow cytometrically by staining with Annexin V-EGFP. Data shown are mean values ± SEM from three independent experiments. Insets serve as illustrations. **b** Cell viability in response to TRAIL (3.55 nM), measured as in (**a**), in the presence or absence of pan-caspase inhibitor QVD-OPH (50 µM). Data show mean values ± SEM from three independent experiments. **c**, **e** 2D-cultivated HCT116 and NCI-H460 cells were detached and HCT116 and NCI-H460 spheroids were dissociated before treatment as in (**a**, **d**). Insets serve as illustrations. **f** The table shows the EC_50_ values determined by nonlinear regressions from (**a**, **c**, **d**, and **e**) (sigmoidal dose response). **g**, **h** Transcript amounts of TRAILR1 and TRAILR2 (long and short isoforms) in 2D-cultivated cells and cells grown as spheroids (day 11), as measured by qPCR. Relative RNA amounts were calculated by the ΔΔCt method using GAPDH for normalization. Data shown are mean values ± SEM from three independent experiments. **i–l** Surface expression of TRAILR1 and TRAILR2. 2D-cultivated cells and cells from spheroids (day 11) were analyzed by flow cytometry. Histograms are representative of at least three independent experiments. Population medians were used to calculate the differences in relative surface expression between 2D- and 3D-cultivated cells. AU arbitrary units. Bar graphs show mean values ± SD of at least three independent experiments. Asterisks indicate statistical significance (*p ≤ 0.05; **p ≤ 0.01; ns not significant; unpaired *t*-test).

We generated spheroids of HCT116 and NCI-H460 cells to study TRAIL responsiveness in 3D culturing conditions. Spheroids reached a diameter of 500–600 µm after 11 days of cultivation and resembled the size and structure of avascular micrometastases, consisting of an outer proliferative cell layer surrounding largely quiescent cells (Supplementary Fig. 1a, b). Compared with HCT116 cells cultured in 2D, HCT116 spheroids were substantially more resistant to TRAIL (Fig. 1a, f). Experiments with pan-caspase inhibitor Q-VD-OPH (QVD) verified that spheroid cells died by apoptosis (Fig. 1b*)*. Interestingly, dissociating the spheroids prior to treatment not only eliminated the increased TRAIL resistance but surprisingly revealed that sizeable subpopulations of cells with an increased TRAIL sensitivity must exist within spheroids (Fig. 1c, f). Similar findings were made in NCI-H460 cells (Fig. 1d–f). Together, these data suggest that TRAIL-hypersensitive cells are protected from cell death within intact spheroids, likely due to TRAIL-resistant cells in outer layers of the spheroids.

Next, we investigated if increased TRAIL resistance in intact spheroids correlates with changed expression of apoptosis regulators in 3D growth conditions. Notably, transcript amounts for both TRAILR1 and R2 dropped when comparing 2D- and 3D-cultured HCT116 and NCI-H460 cells (Fig. 1g, h). Correspondingly, average cell surface amounts of these receptors likewise decreased, with the exception of TRAILR2 in HCT116 cells (Fig. 1i, j). Histogram analyses identified that the entire populations of HCT116 and NCI-H460 cells lose TRAILR1 expression (Fig. 1k, l). HCT116 cells expressed only low amounts of TRAILR2, with a substantial proportion of the population overlapping with the negative control in both 2D and 3D growth scenarios (Fig. 1k). In NCI-H460 cells, TRAILR2 surface amounts dropped considerably, however, the right shoulder of the distribution indicated that a subpopulation of cells still retains TRAILR2 surface expression in amounts at least as high as in the 2D growth scenario (Fig. 1l). Other tested apoptosis regulators, among them DISC components such as FADD and procaspase-8 as well as procaspases-9 and -3 and their inhibitor XIAP, did not change in their expression between 2D and 3D growth conditions. In contrast, cFlip isoforms were downregulated in spheroids. However, the downregulation of these antiapoptotic proteins cannot be accountable for the overall increased TRAIL resistance of spheroids (Supplementary Fig. 2).

Together, these data suggest that a population of TRAIL-hypersensitive cells is protected from cell death within intact spheroids, likely due to TRAIL-resistant cells in outer layers of the spheroids, and that differences in the expression of TRAILR1 and TRAILR2 might account for the observed changes in TRAIL susceptibility.

### Spatial patterns of TRAILR1/R2 expression correlate with TRAIL responsiveness in tumor cell spheroids

**Fig. 2:**
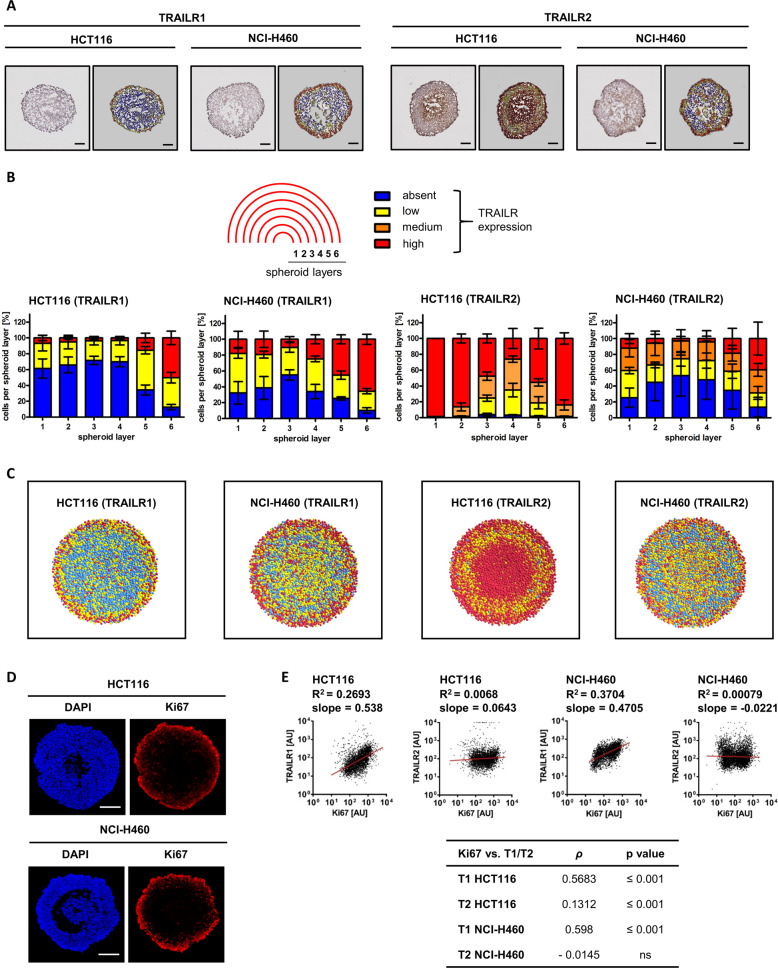
Spatial patterns of TRAILR1/R2 expression correlate with TRAIL responsiveness in tumor cell spheroids. **a** Spheroid slices (day 11) were immunohistochemically stained for TRAILR1 or TRAILR2 and counterstained with hematoxylin. TRAILR staining intensity was color coded (absent (blue), low (yellow), medium (orange), and high (red)). Scale bars = 100 μm. **b** TRAILR expression in spheroid layers. Percentage of cells with no, low, medium, and high TRAILR expression are shown are mean values ± SEM from *n* = 3 spheroids. **c** Representative spheroid cross-sections were reconstructed based on mean spheroid metrics (spheroid diameter, cell number, TRAILR expression amounts) and color coded as in (**a**) and (**b**). **d** Spheroids slices (day 11) were fixed and stained for Ki67. Nuclei were stained with DAPI. Images are representative of three independent experiments. Scale bars = 100 µm. **e** Cells isolated from spheroids (day 11) were flow cytometrically analyzed for surface TRAILR amounts and, following permeabilization, Ki67. Scatter plots are representative of three independent experiments. *R*^2^ were obtained for linear regression. Table shows Spearman’s rank correlation coefficient (*ρ*) rho and associated probability values.

If changes in TRAILR1 and TRAILR2 surface amounts alter TRAIL responsiveness in 3D growth conditions, it would be expected that their expression patterns in spheroid cross-sections correlate with high TRAIL responsiveness in outer and inner but not in intermediate cell layers. We therefore immunohistochemically stained medial sections of HCT116 and NCI-H460 spheroids for TRAILR1 and TRAILR2, and color coded the spheroid sections according to absent, low, medium, and high receptor expression (Fig. 2a). Quantification of receptor amounts in spheroid layers, from the inside to the spheroid surface, provided evidence matching our expectations. TRAILR1 expression was highest in the outermost spheroid layers (HCT116 spheroids: layer 6; NCI-H460 spheroids: layers 5 and 6) and absent or low in the innermost layers (Fig. 2b). In HCT116 spheroids, 80% of all cells in the outermost layer and 80–100% of all cells in the two innermost spheroid layers displayed high TRAILR2 amounts (Fig. 2b). In contrast, the percentage of cells with high TRAILR2 expression in the intermediate layers was substantially reduced. Qualitatively similar results were obtained for NCI-H460 spheroids (Fig. 2b). The antibodies used did not cross react between TRAILRs, ensuring specificity of the obtained signals (Supplementary Fig. 3a, b). From the pooled TRAILR expression data, we reconstructed representative spheroids to more intuitively visualize spatial TRAILR expression patterns (Fig. 2c). Besides high expression of both TRAILRs in the outer spheroid layers, high amounts of TRAILR2 were found in spheroid centers, in particular in the vicinity of dead cores that begin to form in older spheroids. We independently confirmed these results by flow cytometry, co-staining cells for TRAILRs and for Ki67 as a marker for proliferating cells in outer spheroid cell layers (Fig. 2d). The expression of Ki67 and the abundance of TRAILR1 strongly correlated (Fig. 2e), whereas surface TRAILR2 expression was the highest in those cells displaying the strongest and the weakest Ki67 signals, respectively (Fig. 2e). High expression of TRAILR1/R2 in the outermost layers also correlated with caspase-3 processing being limited primarily to these layers in spheroid cross-sections (Supplementary Fig. 3c, d).

Together, these data reveal a cross-sectional pattern of high TRAILR expression in surface layers, followed by low expression or the absence of TRAIL receptors in intermediate layers, and finally high TRAILR2 expression in the innermost layers of tumor cell spheroids.

### TRAILR2 expression is essential for TRAIL hypersensitization within tumor cell spheroids

**Fig. 3:**
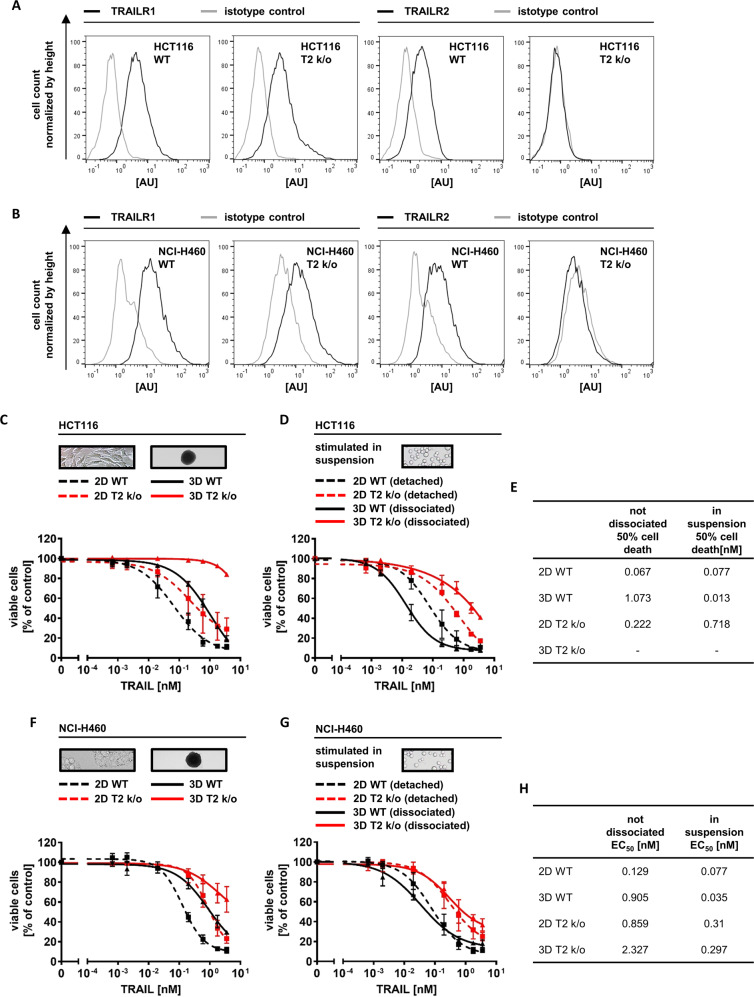
TRAILR2 expression is essential for TRAIL hypersensitization within tumor cell spheroids. **a**, **b** TRAILR surface expression. HCT116/NCI-H460 wildtype (WT) and HCT116/NCI-H460 TRAILR2 knockout (T2 k/o) cells were analyzed by flow cytometry. Histograms are representative of two independent experiments. **c**, **d**, **f**, **g** HCT116/NCI-H460 cells or HCT116/NCI-H460 TRAILR2 knockout (T2 k/o), grown in 2D or as spheroids (day 11) were stimulated with TRAIL for 6 h. In (**d**, **g**), cells were detached, or spheroids were dissociated prior to treatment. Loss of viability was measured flow cytometrically by Annexin V-EGFP staining. Data shown are mean values ± SEM from three independent experiments, with exception of HCT116 T2 k/o cells in (**d**), where bars indicate range (*n* = 2). The experiment shown in (**f**) was performed side by side with the experiment shown in supplementary Fig. 5c, thus the curves derived from WT cells (2D and 3D) are identical. The same is true for the data shown in (**g**) and in supplementary Fig. 5d. Insets serve as illustrations. **e**, **h** EC_50_ values obtained from nonlinear regressions (sigmoidal dose response). Minus sign indicates cases in which the EC_50_ could not be calculated.

To study if TRAILR2 is required for TRAIL hypersensitization within tumor cell spheroids, we targeted *TNFRSF10B* by CRISPR/Cas9-mediated gene knockout. The resulting cells lost TRAILR2 surface and overall TRAILR2 protein expression, without significantly affecting the amounts of TRAILR1 (Fig. 3a, b, Supplementary Fig. 4a). When grown as spheroids, the surface expression of TRAILR1 in HCT116 TRAILR2 k/o cells dropped like in parental HCT116 cells (Supplementary Fig. 4b and Fig. 1k). The loss of TRAILR2 only moderately protected HCT116 cells grown in 2D but strongly protected these cells when grown as spheroids (Fig. 3c). While cells from dissociated parental HCT116 spheroids were TRAIL-hypersensitive, HCT116 TRAILR2 k/o cells remained substantially more resistant (Fig. 3d, e). Similar findings were made in NCI-H460 T2 k/o spheroids (Fig. 3f, g, h). To verify that solely TRAILR2 but not TRAILR1 is accountable for the TRAIL-hypersensitivity of cells close to the spheroid center, we also targeted *TNFRSF10A* in HCT116 and NCI-H460 cells (Supplementary Fig. 4a). In contrast to the knockout of TRAILR2 and as expected, knockout of TRAILR1 did not further increase the TRAIL resistance of intact spheroids (Supplementary Fig. 5a, c) since TRAILR1 expression is already largely lost in inner layers of spheroids grown from the parental cells (Fig. 2a–c). Consequently, knockout of TRAILR1 failed to abrogate TRAIL-hypersensitive cell populations in dissociated spheroids (Supplementary Fig. 5b, d, e, f). These results therefore demonstrate that the presence of TRAILR2 but not TRAILR1 is essential for populations of TRAIL-hypersensitive cells to develop within multicellular spheroids.

### Oxygen and nutrient deprivation result in accumulation of TRAILR2

With growing spheroid sizes, microenvironmental stress increases, in particular within the center of avascular cell masses, typically arising from a lack of oxygen and nutrients [15]. Therefore, we next sought to identify which stress factors are linked to the accumulation of TRAILR2 in the center of spheroids.

In older spheroids, excessive stress results in the formation of dead cores. We therefore first tested if TRAILR deregulation can already be observed prior to the formation of dead spheroid centers. We noted that TRAILR2 deregulation begins to manifest on day 7, several days before necrotic cores develop (Supplementary Fig. 6), and therefore cannot be a consequence of a microenvironment altered by dying cells.

**Fig. 4:**
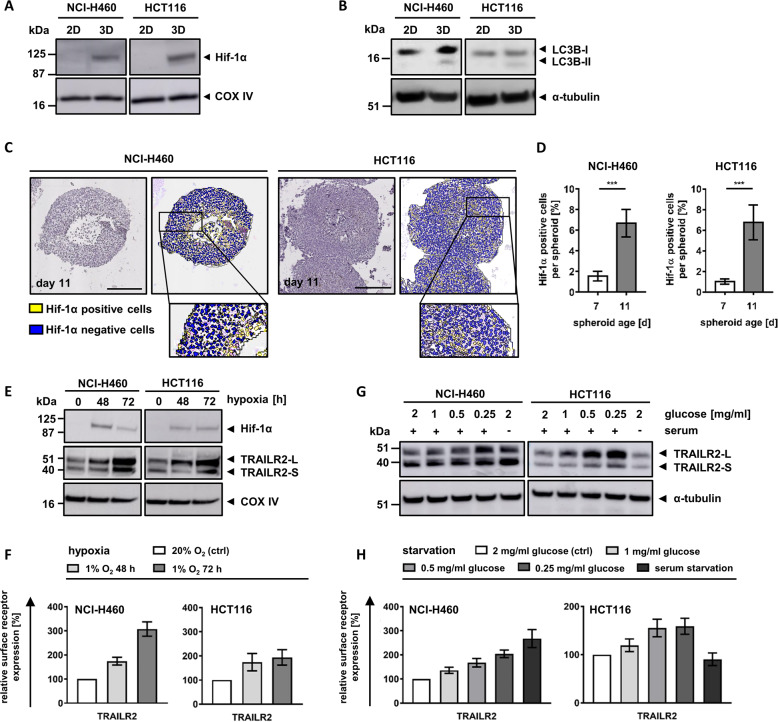
Oxygen and nutrient deprivation result in accumulation of TRAILR2. **a**, **b** Cell lysates of 2D-cultivated cells or cells grown as spheroids (day 11) were analyzed by western blotting. Blots are representative of three independent experiments. **c** Paraffin-embedded spheroid slices (day 11) were immunohistochemically stained for Hif-1α and counterstained with hematoxylin. Cells were color coded as positive (yellow) or negative (blue). Pictures are representative of three independent experiments. Scale bars = 200 μm. **d** Percentages of cells positive or negative for Hif-1α expression are shown as mean values ± SD of 4–16 spheroids from 3 independent experiments. Asterisks indicate statistical significance (***p ≤ 0.001; unpaired *t*-test). **e** Lysates from cells cultivated at 1% O_2_ were analyzed by western blotting. Blots shown are representative of three independently performed experiments. **f** TRAILR2 surface expression measured by flow cytometry. Medians of the cell populations were used to calculate surface expression relative to cells cultivated at control conditions. Data are mean values ± SD of at least three independent experiments. **g** Lysates from cells cultivated at starvation conditions were analyzed by western blotting. Blots shown are representative of three independently performed experiments. **h** TRAILR2 surface expression measured by flow cytometry. Medians of the cell populations were used to calculate surface expression relative to cells cultivated at control conditions. Data are mean values ± SD of at least three independent experiments.

Comparing 2D and 3D growth conditions, we found that cells grown as spheroids induce hypoxia-inducible factor-1α (Hif-1α), a canonical marker for oxygen deprivation (Fig. 4a). Likewise, cells obtained from spheroids presented with altered amounts or balances of lipidated and unlipidated microtubule-associated proteins 1A/1B light chain 3B (LC3B), indicative of nutrient deprivation (Fig. 4b). Stress appeared to manifest primarily within the center regions of spheroids and to increase over time, as shown for Hif-1α accumulation and localization (Fig. 4c, d). To study if TRAILR2 expression is a consequence of these stress factors, we next replicated hypoxia and nutrient deprivation in conventional cell culturing conditions. Hypoxia indeed resulted in a pronounced accumulation of total and surface exposed TRAILR2 in both NCI-H460 and HCT116 cells (Fig. 4e, f). Glucose starvation likewise induced substantial accumulation of TRAILR2 in both cell lines, while serum starvation caused strong accumulation of TRAILR2 only in NCI-H460 cells (Fig. 4g, h).

From these data we conclude that hypoxia and nutrient deprivation, primary stress factors within 3D growth environments, are sufficient to cause TRAILR2 accumulation similar to what is observed within the centers of spheroids.

### COX-II inhibitor celecoxib enhances TRAILR2 expression and synergizes with TRAIL treatment in eliminating cancer cell spheroids

**Fig. 5:**
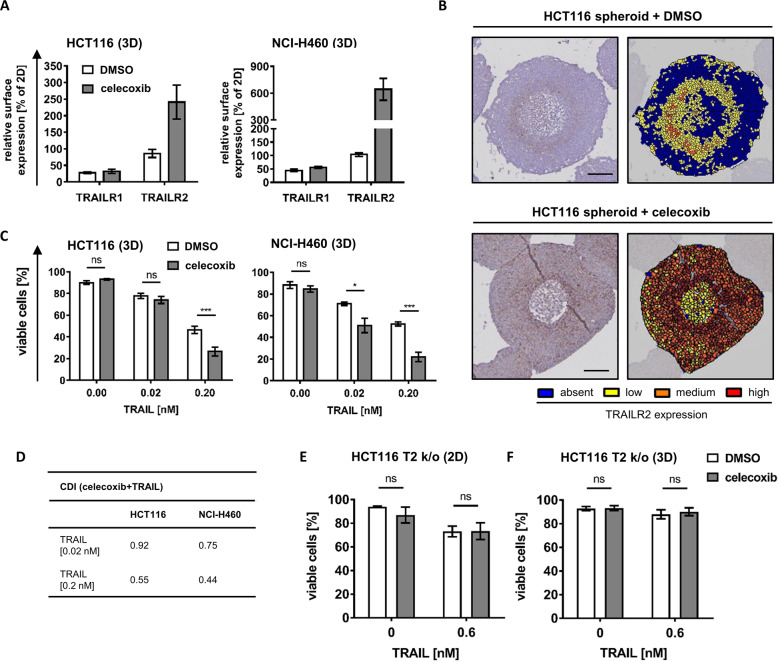
COX-II inhibitor celecoxib enhances TRAILR2 expression and synergizes with TRAIL treatment in eliminating cancer cell spheroids. **a** Cells grown in 2D or as spheroids (day 11) were treated with 50 μM celecoxib for 72 h. TRAILR expression was determined by flow cytometry. Data are shown as mean values ± SEM from at least three independent experiments. **b** HCT116 spheroids (day 11) were stimulated with 50 μM celecoxib for 72 h. Paraffin-embedded slices were immunohistochemically stained for TRAILR2 and counterstained with hematoxylin. Cells were color coded according to TRAILR2 expression amounts (absent (blue), low (yellow), medium (orange) and high (red)). Images are representative of two independent experiments. Scale bars = 100 μm. **c**, **d** Spheroids of HCT116 and NCI-H460 cells (day 11) were stimulated with 50 µM celecoxib for 72 h, with TRAIL added after 48 h. The loss of viability was determined flow cytometrically by Annexin V-EGFP staining. Data show mean values ± SEM of three independent experiments. Asterisks indicate statistical significance (*p ≤ 0.05, ***p ≤ 0.001, ns not significant; Two-way ANOVA with Bonferroni correction). Data from (**c**) served to calculate the coefficient of drug interaction (CDI), with values < 0.7 indicating strong synergism (**d**). **e**, **f** Cells grown in 2D or as spheroids (day 11) were stimualted with 50 µM celecoxib for 72 h, with 0.6 nM TRAIL added after 48 h. Viability loss was determined by Annexin V-EGFP staining and flow cytometry. Data are mean values ± SEM from three independent experiments.

We next studied if the expression of TRAILR2, which is lost or reduced in intermediate spheroid cell layers, can be restored in order to enhance TRAIL responsiveness in 3D growth conditions. Celecoxib is an FDA-approved nonsteroidal anti-inflammatory drug that can enhance TRAILR2 expression in conventionally cultured colon and prostate cancer cells, presumably through inducing ER stress and the expression of CHOP, a major ER stress-induced transcription factor known to promote TRAILR2 expression [17, 18]. Indeed, celecoxib induced CHOP in HCT116 and NCI-H460 cells (Supplementary Fig. 7a) and resulted in pronounced accumulation of TRAILR2 but not TRAILR1 in cancer cell spheroids (Fig. 5a). The expression of DISC components FADD, cFlip or procaspase-8 was not affected by celecoxib treatment (Supplementary Fig. 7b). Analyzing spheroid cross-sections revealed that TRAILR2 expression in the presence of celecoxib was no longer restricted to the outer and innermost spheroid layers but was also found in high amounts in intermediate cell layers (Fig. 5b, c). The upregulation of TRAILR2 indeed substantially sensitized both HCT116 and NCI-H460 spheroids to TRAIL treatment (Fig. 5c), with clear indications for pronounced response synergies for this combination treatment in both cell line models (Fig. 5d). On the contrary, stimulating 2D-cultured HCT116 TRAILR2 k/o cells and HCT116 TRAILR2 k/o spheroids with celecoxib failed to sensitize the cells to TRAIL, indicating that after the combination treatment cells died due to an increase in TRAILR2 expression (Fig. 5e, f). Celecoxib treatment therefore seems sufficient to increase TRAILR2 expression in intermediate, otherwise TRAIL-resistant spheroid layers, offering a tangible opportunity to improve responsiveness to TRAIL-based therapeutics.

## Discussion

Here, we identified that pronounced, spatially coordinated TRAIL response heterogeneities manifest within cancer cell spheroids, leading to the development of TRAIL-resistant cell layers that enclose TRAIL-hypersensitive cells. Reduced TRAIL responsiveness arises from the loss of TRAILR1 and R2 expression. Importantly, microenvironmental stress, such as nutrient and oxygen deprivation, together with pharmacologically induced ER stress, are sufficient to restore TRAILR2 expression and treatment responsiveness.

While TRAILRs are preferentially expressed in cancer cells and are a prerequisite for TRAIL responsiveness [19–22], surprisingly little is known about the heterogeneity of TRAILR expression within individual tumors. Corresponding to our findings in the outer proliferative layers of spheroids, the invasive fronts of colon tumors express high amounts of TRAILR2 [23]. Indeed, proliferation and invasion of various cancers might at least in part depend on autonomous TRAILR signaling. For example, it was recently shown that TRAIL/TRAILR2 signaling increases migration and invasion via a Rac1/PI3K signaling axis in KRAS mutated nonsmall-cell lung cancer and pancreatic ductal adenocarcinomas [24]. Additional “non-canonical” signaling upon activation of TRAILRs likewise promotes cell proliferation, including the activation of the NF-*κ*B pathway as well as JUN kinase and MAP kinase signaling [25–29]. In addition to its cell surface receptor function, nuclear TRAILR2 suppresses the maturation of miRNA let-7 and thereby promotes the proliferation of pancreatic cancer cells [30]. While TRAILR expression obviously is essential for apoptosis induction by TRAILR agonist-based therapeutics, tumor autonomous nonapoptotic signaling through TRAILRs is associated with poor patient outcome in current clinical settings. For example, TRAILR2 expression is associated with poor prognosis in pancreatic cancer and KRAS mutated colon cancer [24]. Similarly, high amounts of TRAIL prognosticate poorer outcome of patients with stage II and III colorectal cancer [31]. We showed that cells close to dead cores of cancer cell spheroids are hypersensitive to TRAIL, and that this hypersensitivity requires the presence of TRAILR2. These cells appear to experience substantial microenvironmental stress, due to oxygen and nutrient deprivation. The absence of glucose enhances TRAILR2 transcription and protein accumulation in HeLa cells as a consequence of ER stress [32], as does treatment with 2-deoxy-D-glucose, an antimetabolic glucose derivative [33]. Hypoxia-induced TRAILR2 expression has likewise been described, but this response seems to differ notably between cell lines [34]. Regarding the reasons underlying the accumulation of TRAILR2 in cells close to spheroid cores it is therefore conceivable that a prolonged and extensive oxygen and nutrient deprivation results in robust induction of ER stress and in the activation of the unfolded protein response. Interestingly, besides elevated cell surface amounts of TRAILR2, this TRAIL receptor can also accumulate intracellularly in response to ER stress and thereby contribute to ligand independent but caspase-8-dependent apoptosis [32, 35–37] Correspondingly, directly inducing persistent ER stress by thapsigargin promotes TRAILR2 accumulation and apoptotic cell death [36]. Even though cells deprived of oxygen and nutrient within the centers of spheroids or micrometastases ultimately will die, it could be speculated that elevated TRAILR2 expression close to spheroid cores contributes to this being an ordered cell death process. The presence or absence of TRAILR2 and apoptosis as a route toward a subsequently phenotypically necrotic core might substantially affect microenvironmental inflammatory signals and cytokine profiles, and by extension the ER stress-dependent immunogenicity of cell death within spheroids [38–40]. Interestingly, cells at the interface of proliferative and quiescent spheroid layers loose both TRAILR1 and R2 expression and thereby reduce the overall TRAIL responsiveness of intact spheroids. TRAILR deficient cells reside in regions in which microenvironmental stress would be considered moderate. Nevertheless, this seems sufficient for TRAILR deregulation, as also supported by studies investigating scenarios of mild, nonlethal stress. For example, limiting oxygen supply drives PI3K signaling [41], which in turn can support hedgehog signaling and expression of GLI3, a repressor of TRAILR1 transcription [42–44]. Furthermore, the mRNA binding protein HuR was suggested to inhibit TRAILR2 translation under such conditions [45]. Since the ER folding and glycosylation capacity depends on nutrient supply, it is noteworthy that under moderate ER stress TRAILR2 mRNA is degraded via IRE1α signaling [36]. Overall, it is therefore conceivable that at conditions of mild deprivation of nutrients or oxygen, not only proliferation slows down but also TRAILR expression is downregulated, thereby increasing apoptosis resistance. The mechanistic basis for this, however, seems highly complex.

We succeeded in counteracting the development of TRAIL-resistant cell layers by intensifying microenvironmental stress by ER stress inducer celecoxib. Celecoxib is an FDA-approved, nonsteroidal anti-inflammatory drug, targeting COX-II but also evoking COX-II independent ER stress [46, 47]. Both COX-II inhibition as well as COX-II independent ER stress potently induce TRAILR2 expression [17, 18, 48–50]. However, TRAIL sensitization by celecoxib was never studied in 3D growth scenarios. We here demonstrate that treatment with celecoxib results in enforced TRAILR2 expression in all spheroid layers, accompanied by increased TRAIL sensitivity. Celecoxib could therefore become an attractive co-treatment option in future TRAIL-based therapies for solid tumors. Besides TRAIL sensitization, antagonizing COX-II-linked cell proliferation, tumor vascularization, and metastasis might provide additional benefits [51–57].

Overall, our study provides novel and detailed insight into how TRAIL response heterogeneities manifest within well-defined multicellular environments, and how cells within these environments can be manipulated to minimize or eliminate barriers of TRAIL resistance.

## Supplementary information

supplemental figure 1

supplemental figure 2

supplemental figure 3

supplemental figure 4

supplemental figure 5

supplemental figure 6

supllemnetal figure 7
