# Supplementary figures and images for: Stress-induced TRAILR2 expression overcomes TRAIL resistance in cancer cell spheroids
Source: Cell Death Differ. 2020 May 20;27(11):3037–52. doi: 10.1038/s41418-020-0559-3 (PMC7560834; doi:10.1038/s41418-020-0559-3)

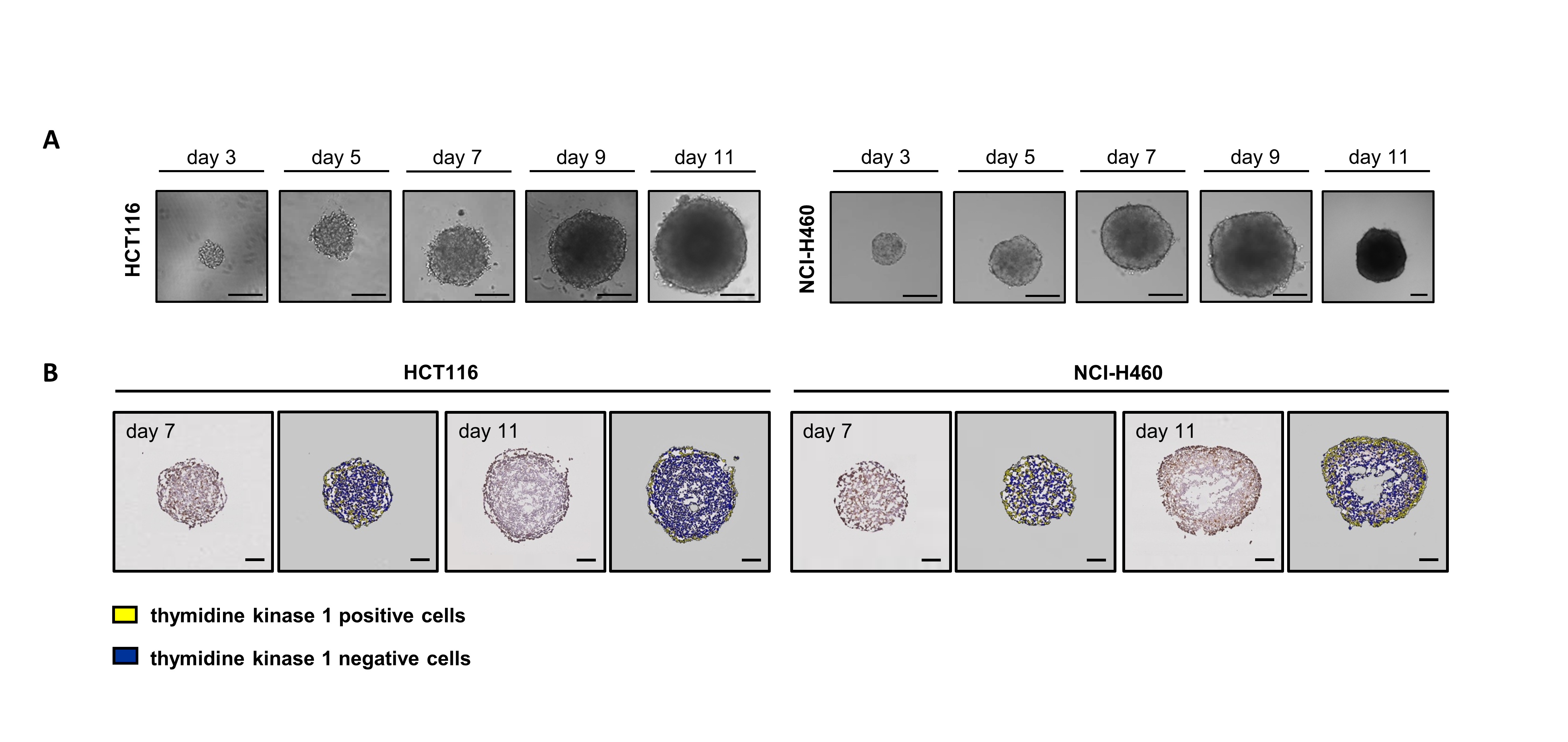

Supplement: Supplementary file 1 — supplemental figure 1 [file 41418_2020_559_MOESM1_ESM.tif]

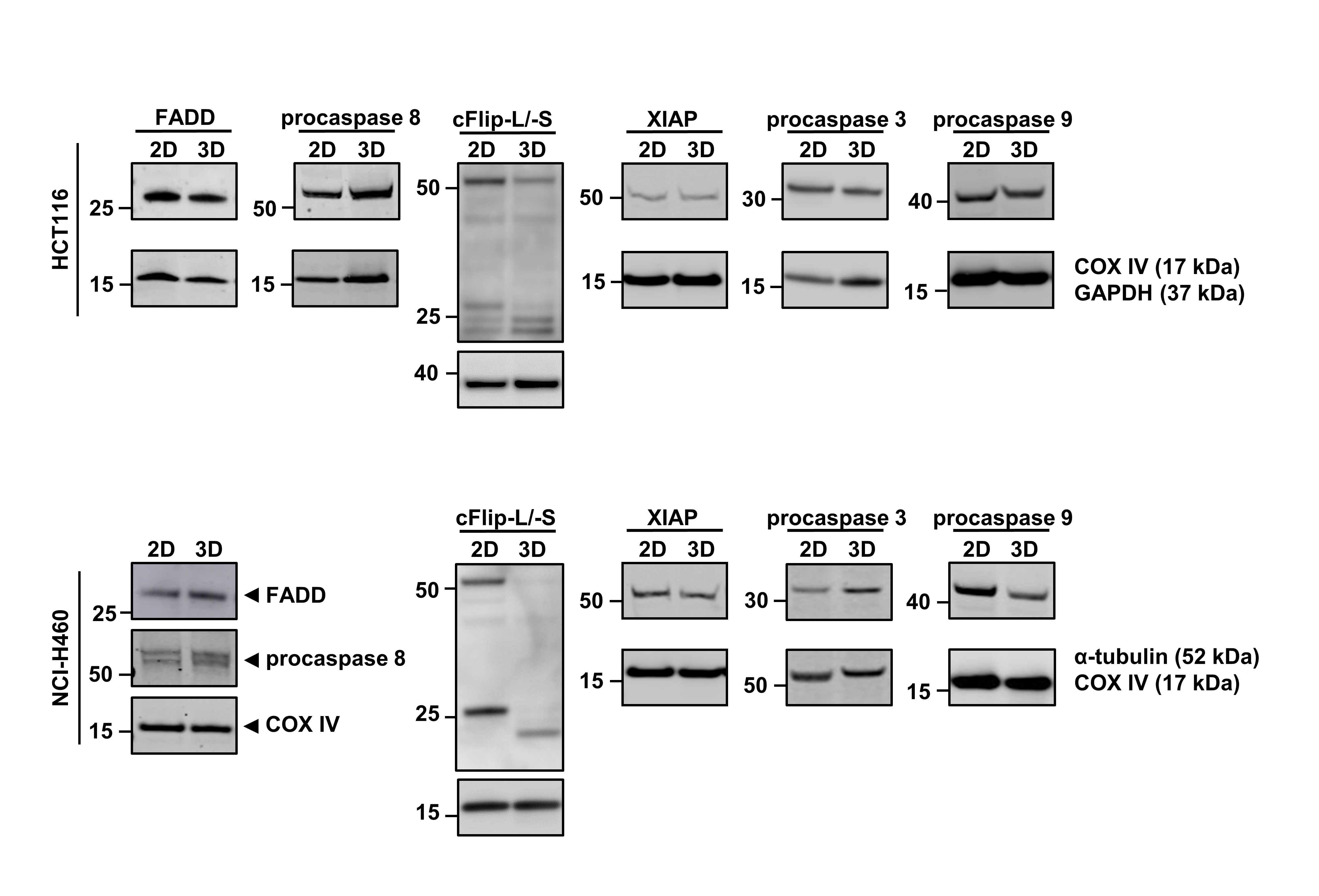

Supplement: Supplementary file 2 — supplemental figure 2 [file 41418_2020_559_MOESM2_ESM.tif]

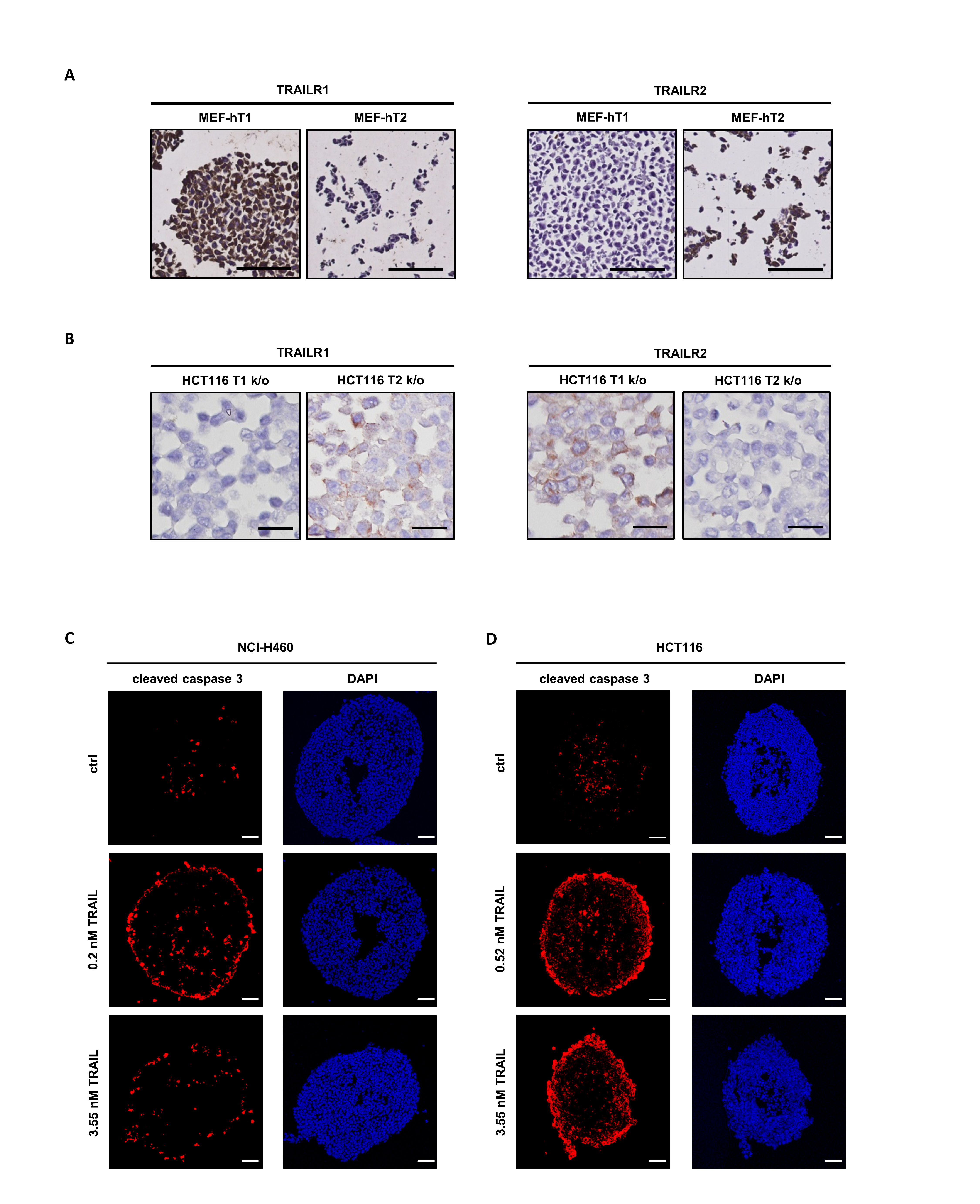

Supplement: Supplementary file 3 — supplemental figure 3 [file 41418_2020_559_MOESM3_ESM.tif]

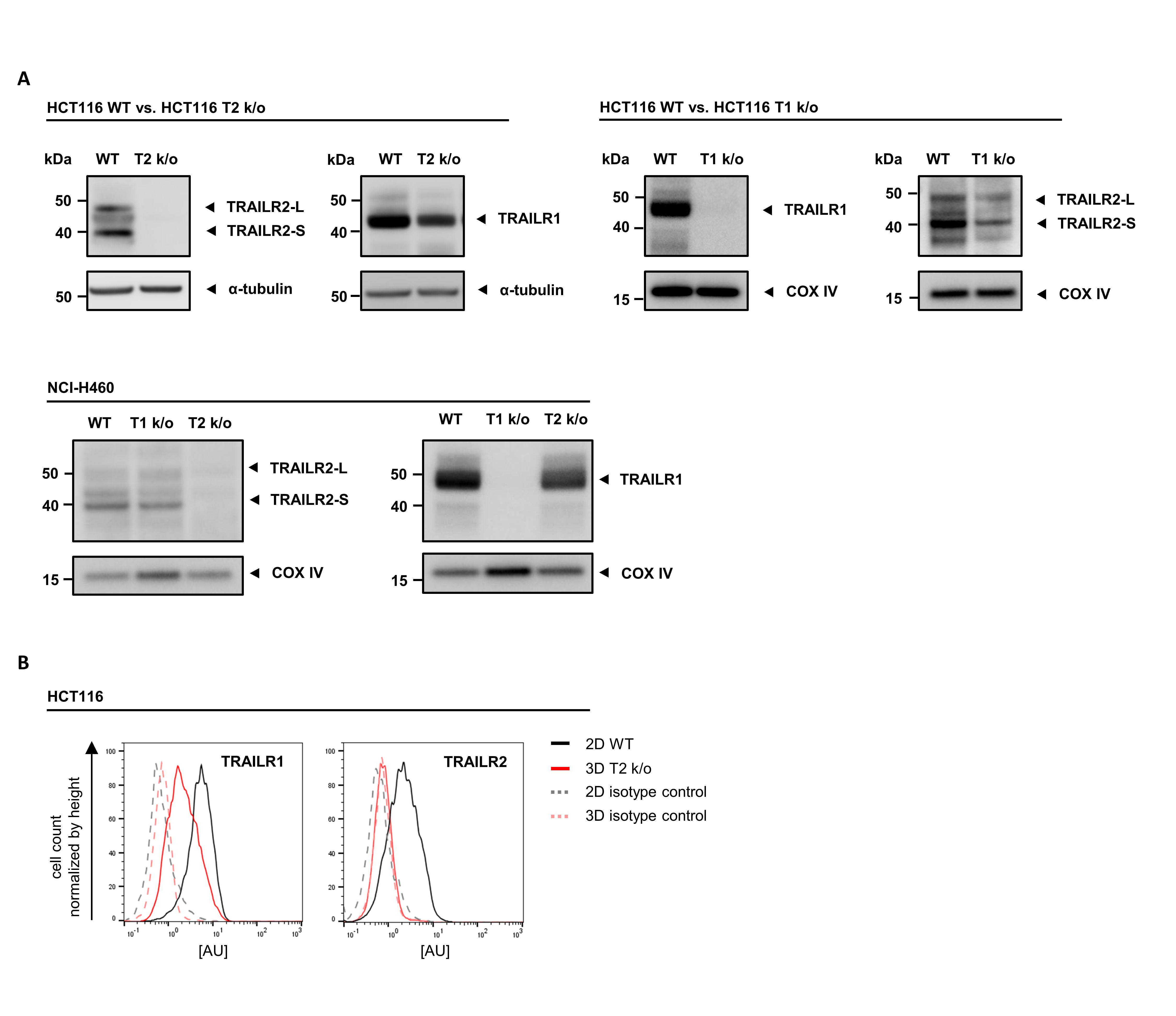

Supplement: Supplementary file 4 — supplemental figure 4 [file 41418_2020_559_MOESM4_ESM.tif]

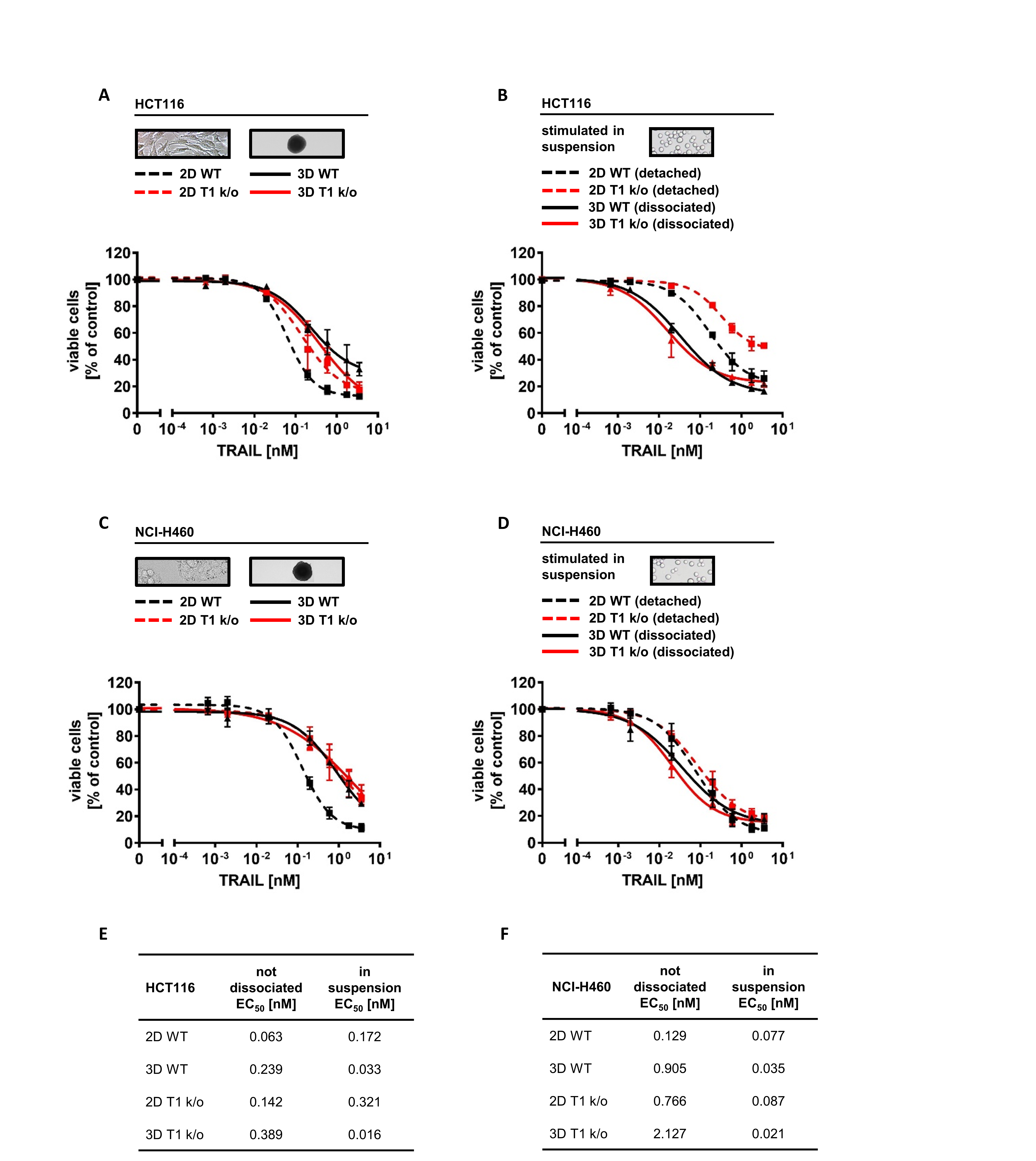

Supplement: Supplementary file 5 — supplemental figure 5 [file 41418_2020_559_MOESM5_ESM.tif]

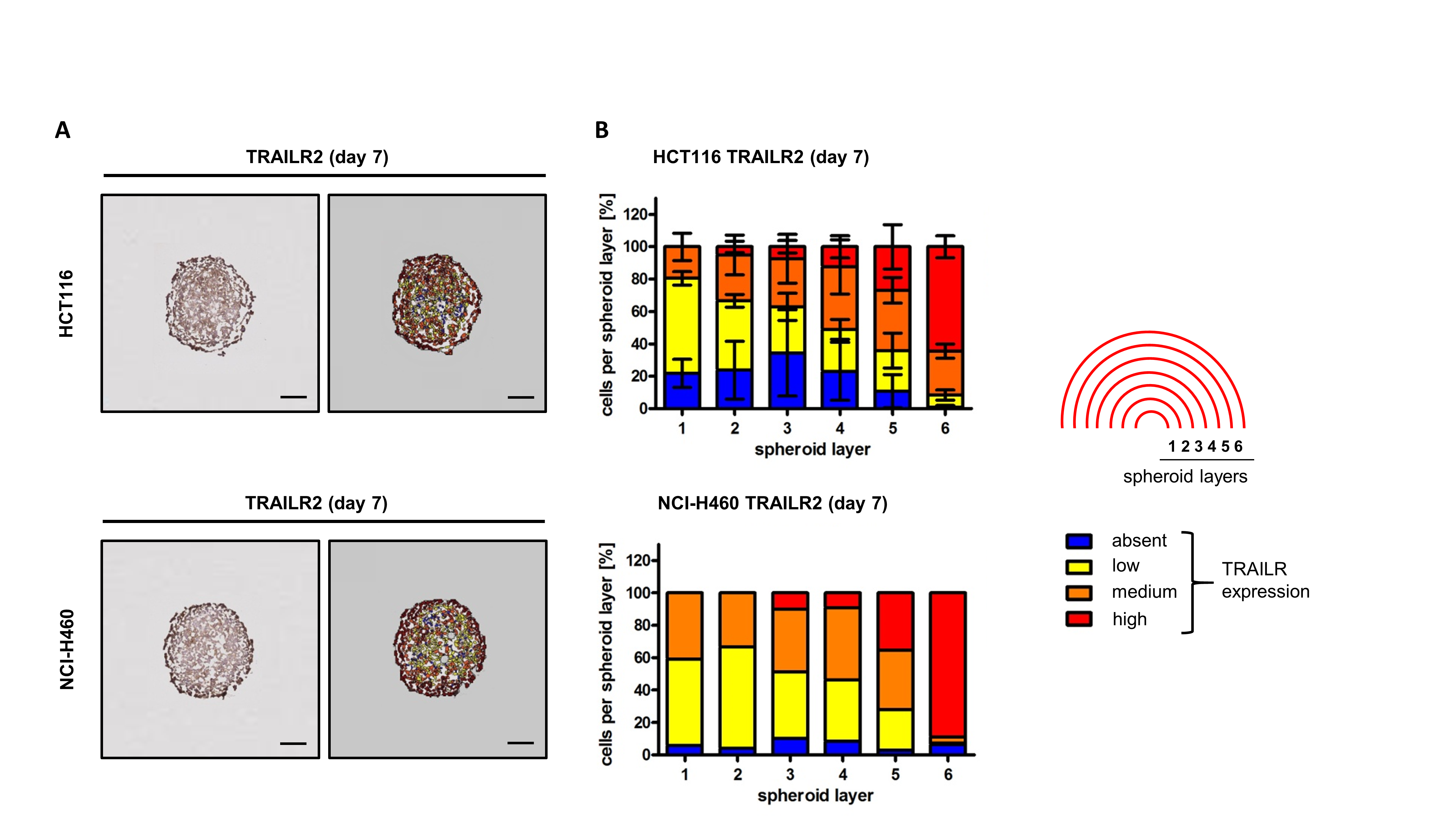

Supplement: Supplementary file 6 — supplemental figure 6 [file 41418_2020_559_MOESM6_ESM.tif]

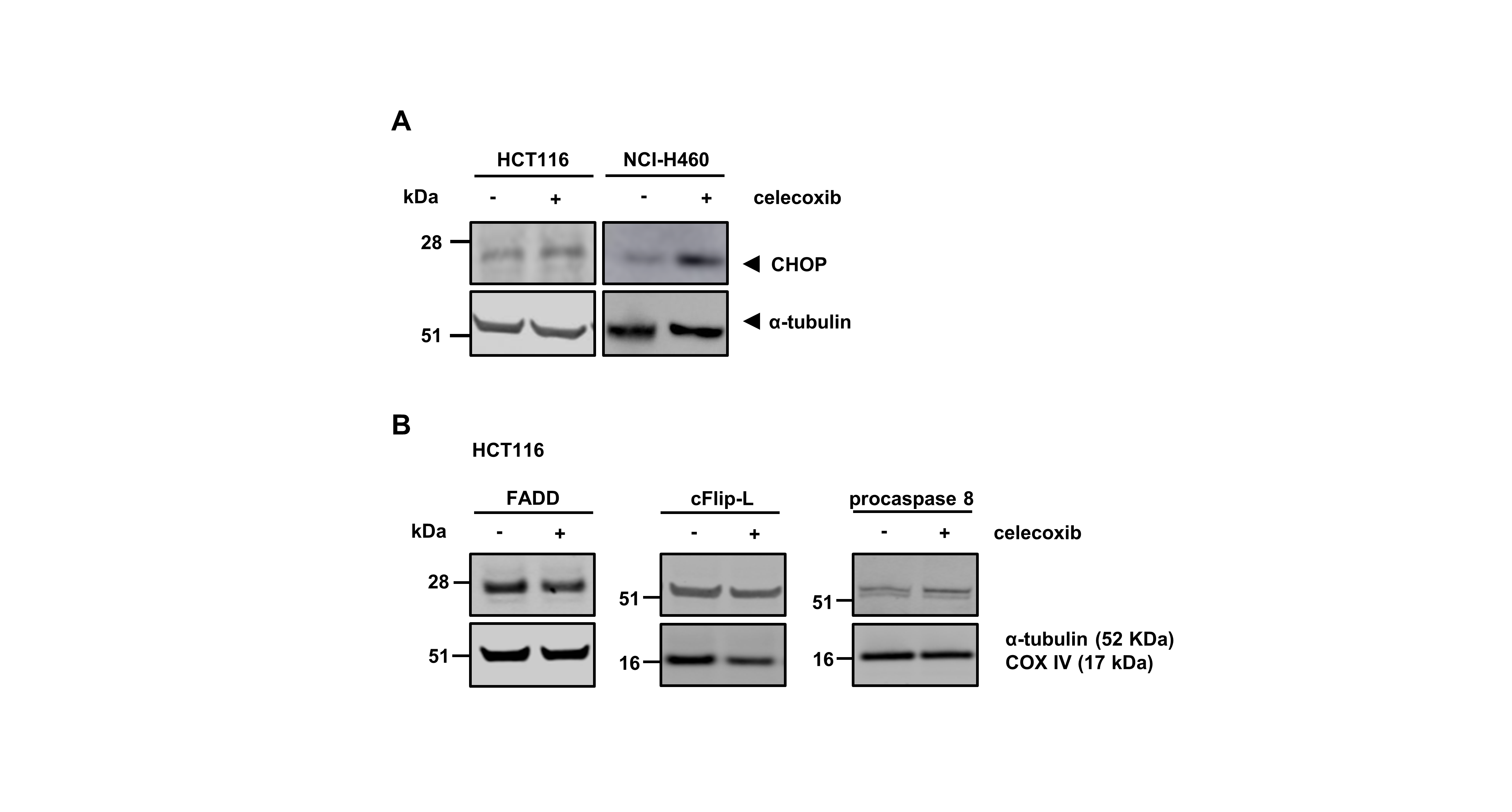

Supplement: Supplementary file 7 — supllemnetal figure 7 [file 41418_2020_559_MOESM7_ESM.tif]
